# Type and magnitude of non-compliance and adulteration in neroli, mandarin and bergamot essential oils purchased on-line: potential consumer vulnerability

**DOI:** 10.1038/s41598-021-90307-2

**Published:** 2021-05-27

**Authors:** Marissa Pierson, Xavier Fernandez, Sylvain Antoniotti

**Affiliations:** 1 European Research Institute on Natural Ingredients (ERINI), 4 Traverse Dupont, 06130 Grasse, France; 2 Université Cote d’Azur, CNRS, Institut de Chimie de Nice, Parc Valrose, 06108 Nice Cedex 2, France; 3 Université Cote d’Azur, Institute for Innovation and Partnerships in Flavor, Fragrance, Cosmetics, Grasse Biotech, 45 Bd Marcel Pagnol, 06130 Grasse, France

**Keywords:** Chemical safety, Analytical chemistry, Natural products

## Abstract

Thirty-one samples of essential oils used both in perfumery and aromatherapy were purchased to business-to-consumers suppliers and submitted to standard gas chromatography-based analysis of their chemical composition. Their compliance with ISO AFNOR standards was checked and revealed, although ISO AFNOR ranges are relatively loose, that more than 45% of the samples analyzed failed to pass the test and more than 19% were diluted with solvents such as propylene and dipropylene glycol, triethyl citrate, or vegetal oil. Cases of non-compliance could be due to substitution or dilution with a cheaper essential oil, such as sweet orange oil, blending with selected compounds (linalool and linalyl acetate, maybe of synthetic origin), or issues of aging, harvest, or manufacturing that should be either deliberate or accidental. In some cases, natural variability could be invoked. These products are made available to the market without control and liability by resellers and could expose the public to safety issues, in addition to commercial prejudice, in sharp contrast with the ever-increasing regulations applying to the sector and the high demand of consumers for safe, controlled and traceable products in fragrances and cosmetic products.

## Introduction

Essentials oils (EOs) are complex natural substances obtained by physical treatments of selected parts of plants. In most instances, distillation is used, either through hydro-distillation techniques or, for more sensitive materials, by steam distillation. In the particular case of the *Citrus* genus, cold pressure is generally applied to the pericarp to obtain the EO^1^.

The fraction of the metabolome of plants as observed in EOs is mostly composed of terpenes, terpenoids, and phenylpropanoids. Sometimes, linear hydrocarbons are also observed as in rose EOs, or other compounds with sulphur-containing functional groups such as thiols or thiocyanates. Considering the method of obtention, EOs are almost exclusively volatile materials, although in the case of cold-pressed EOs, a non-negligible fraction can be non-volatile.

As volatile material, the analytical technique of choice for EOs is gas chromatography (GC) which allows for, when conducted with adequate and validated methods, both qualitative and quantitative characterization^1^. Typically, identity of analytes is obtained by coupling mass spectrometry (MS) with gas chromatography (GC–MS), determining retention indexes and co-injecting pure compounds if necessary, while their proportion in the mixture is determined by using a stable and universal detector such as a flame ionization detector (GC-FID) using the corrected response factor method^2,3^. The chemical composition of EOs thereby obtained is reproducible, and only subjected to natural variation that could occur depending on geographical, seasonal, or agricultural factors and manufacturing processes^4^.

For centuries EOs have attracted the attention of mankind for their biological properties, either as odoriferous material for religious or pagan practice, and perfumery, or for therapeutic applications, either in folk medicine, aromatherapy or pharmaceutical activities^5,6^. As potentially biologically active material, EOs sometimes contain significant quantities of toxic compounds such as methyleugenol (suspected carcinogen)^7,8^, safrole (weak hepatocarcinogen among other adverse effects), estragole (suspected carcinogen and genotoxic)^9–13^, furocoumarins (dermatitis inducers)^14,15^, and allergenic compounds, among others^16^.

**Figure 1:**
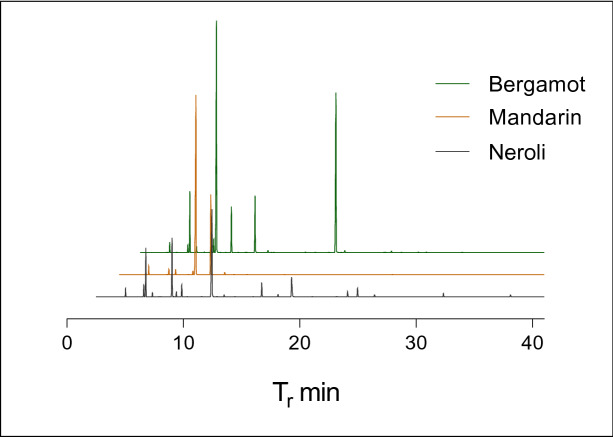
Examples of total ion chromatograms, offset by 1 min each, of *Citrus* essential oils (bergamot, mandarin, and neroli essential oils). Chemical compositions are detailed below.

In the particular case of *Citrus* EOs, the chemical composition features mostly monoterpenes and monoterpenoids, the prominent metabolite being limonene, either as the *R* or *S* enantiomer depending on the genus, species or even cultivar^17,18^. *Citrus* EOs such as bergamot (*Citrus bergamia*), mandarin (*Citrus reticulata*), or neroli (*Citrus aurantium* ssp Amara or Bigaradia, from which only the flowers are steam distilled) EOs play an important role in fine perfumery for their delicate scents; while sweet orange (*Citrus sinensis*) EO, which is almost entirely constituted of (*R*)-limonene, has very little olfactory interest (Fig. 1).

In vitro and in vivo biological activities for *Citrus* EOs have been reported such as antimicrobial, antiviral and antioxidant properties, insect repellent activities^19^, as well as effects on central nervous system (anxiety, attention, relaxation, sleep, mood, stress …)^20^ and should be used as natural preservatives^21–23^.

**Figure 2:**
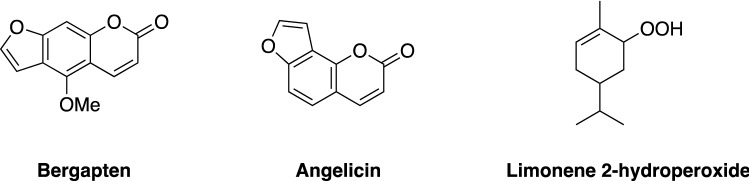
Chemical structures of some furocoumarins and limonene 2-hydroperoxide.

On a darker side, toxicity issues arise in the presence of furocoumarins, present in the non-volatile fraction of some *Citrus* EOs obtained by cold-pressure along with the EO (vide supra), and hydroperoxides, such as limonene hydroperoxide due to aging and auto-oxidation under air and light exposure^24^. Furocoumarins and hydroperoxides can induce skin disorders such as contact dermatitis and other allergenic reactions, and in worse cases exhibit photogenotoxicity for the former (Fig. 2)^14^. Furocoumarins total content should not exceed 1 ppm in cosmetic products in Europe^25^.

EOs can be obtained from various part of most plants (flowers, leaves, steams, buds, roots, bark …) resulting in a large diversity of EOs. However, because of the similarities in the early steps of plant metabolism, individual compounds such as monoterpenes are found in the essential oils of many plants. In the case of sweet orange EO, huge quantities are available on the market because it is a co-product of orange juice manufacture resulting in low cost and large availability (30,000 tons in 2004 and increasing)^26^. At the same time, rare EOs of high demand and/or of limited offer such as those obtained from rose (*Rosa damascena*), agarwood (*Aquilaria malaccensis*), orris (*Iris germanica*) or ylang ylang (*Cananga odorata*) are difficult to find and their value can reach several thousands of euros per kilogram. This contrast has resulted in economically motivated adulteration (EMA), a fraudulent practice consisting in adding cheap material to an expensive one to increase profit^27^. Adulteration of essential oils is typically observed in four different types:

- Dilution with a solvent featuring similar physico-chemical properties to EOs such as vegetal oils, or organic solvents (triethylcitrate, (di)propylene glycol, alkyl phthalates, …);
- Addition of a cheaper EO more or less close in terms of origin (same genus) or chemical composition (e.g. sweet orange EO (*Citrus sinensis*) in other *Citrus* EOs or palmarosa EO (*Cymbopogon martini*) in rose EOs);
- Addition of individual compounds, either natural or synthetic, to mimic the olfactory properties, the composition or the chemotype (e.g. linalyl acetate to mimic lavender EO (*Lavendula angustifolia*) or neryl acetate to mimic immortelle EO (*Helichrysum italicum*));
- Substitution with another EO or mixture of EOs of low value (sweet orange oil, turpentine oil, …) to which key compounds, either natural or synthetic, are added.

These types of deliberate practice should not be agglomerated with accidental events (errors of botanical authentication, inadvertent blending of vegetal material, sometimes handled as powders, contamination during manufacture, storage, labelling, …) or natural variation that result in non-compliance. Trading of EOs is framed by normative references such as ISO and AFNOR providing industry accepted ranges of concentration for selected compounds in each EO^28^.

In all these cases, analytical chemistry is required to characterize adulteration both qualitatively and quantitatively^29^. Adulteration techniques have evolved continuously along with analytical countermeasures to reach a high level of sophistication. Indeed, besides sensory and physico-chemical properties such as density, optical rotation, or refractive index, R&D and QC labs have developed techniques and combinations of techniques to identify even the most subtle cases of adulteration. Worth mentioning but not limited to are isotopic approaches (^13^C/^12^C ratios or ^14^C contents)^30^, enantioselective analyses^31^, multidimensional GC techniques^32^, or spectroscopic or spectrometric methods (NMR^33^, fluorescence^34^, IR^35^, Raman^36^) coupled with metabolomic approaches.

In contrast with these accurate, efficient, and sensitive techniques in constant evolution which enable professionals to detect most cases of adulteration, consumers in business to consumer (B to C) commerce have limited knowledge about issues of adulteration and no analytical capabilities and can only count on the fairness of their suppliers. The inherent risk for consumers handling concentrated, potentially biologically active, molecules with cases of overdose, misuse and intoxication in family practice is compounded upon when these products may not match their labels^37^.

In this context, we embarked into a study of the quality of EOs purchased on-line directly from EOs suppliers web sites or from global shopping platforms operating worldwide. We have chosen to focus on three *Citrus* EOs: mandarin (*Citrus reticulata*), bergamot (*Citrus bergamia*), and neroli (*Citrus aurantium* ssp Amara or Bigaradia).

## Results and discussion

In the frame of a research program dedicated to the study of authenticity and naturality of essential oils, and considering the increasing interest of consumers for essential oils, natural products, home-made cosmetics and household products, we became interested in sampling essential oils purchased on-line to evaluate their quality. We thus purchased samples of EOs of mandarin (*Citrus reticulata*), bergamot (*Citrus bergamia*), and neroli (*Citrus aurantium* ssp Amara or Bigaradia). With 23 samples out of ca. 230 made available from a large platform operating on-line, the sampling size was deemed sufficient to have a first estimate (± 20% at the 95% level of confidence, for a normal distribution). To these 23 samples were added 6 additional samples purchased from other retail sources, mostly online as well, and 6 references. To encompass the largest variability possible, we decided to simply start with checking the compliance of these samples with ISO norms using gas chromatography coupled with flame ionization detector (FID) and mass selective analyzer (MS).

### Analysis of neroli essential oil (NEO)

Nine commercial samples of NEO were analyzed and their peak areas were compared to ISO Norm 3517^38^. Two samples were acquired from a reputable source and considered premium samples. Of the nine neroli samples, only one sample, N4, met all of the ISO chromatographic specifications. The ISO standard includes acceptable ranges for 13 compounds; α-pinene, β-pinene, sabinene; β-myrcene, limonene, (*E*)-β-ocimene, linalool, α-terpineol, linalyl acetate, neryl and geranyl acetate, (*E*)-nerolidol and (*E*,*E*)-farnesol.

Samples N6 and N9, were not compliant with all 13 compounds in the ISO range. N9 is low in β-pinene and high in linalyl acetate at 16.19 ± 0.02% compared to the 3–15% range. N6 had 21.42 ± 0.02% linalyl acetate, significantly outside the ISO range.

N1 and N2 were purchased online from two different labels but shared nearly identical compositions. Both had low values for β-pinene, β-myrcene, limonene, linalool and (*E*)-nerolidol. Linalyl acetate constituted over 26% of the oils’ compositions; more than 10% higher than the ISO prescription. Sabinene and α-pinene were not identified in either sample, however the standard range extends to traces of these compounds. Comparing the peak areas to the premium samples shows a gross adulteration by dilution, likely with a vegetal oil or propylene glycol type diluent.

N3 and N4 were two of the best samples compared to the ISO requirements. N3 had low β-myrcene and (*E*)-nerolidol content. N4 met all of the prescribed values. N3’s adherence is surprising due to its price point at less than $1/mL compared to N4’s $5.32/mL. This is not to say there is no chance of adulteration in either of these samples, merely that these samples meet, or nearly meet, the advice of the ISO reference.

N5 fell out-of-range for 6 out of the 13 compounds.

N7 and N8 were severely adulterated with di-propylene glycol and propylene glycol, respectively. The chromatograms clearly show the adulterants as well as the resulting peak reduction of the compounds of interest (see S.I.). The coelution of the adulterant and the analyte peaks makes it difficult to even compare the samples to the ISO reference. This disregard toward the obviousness of adulteration is indicative of the fact that consumers have no real means of verifying the quality of the product. The business knows that the consumer has no access to GC and can adulterate without sophistication.

In addition, to boost olfactory properties, N7 contained 4-tert-butylcyclohexyl acetate (mixture of diastereoisomers), a well-known synthetic woody odorant, and N1, N2 and N7 contained nerolin and methyl-β-naphtyl ketone, which are synthetic materials known for decades exhibiting neroli perfume^39^.

**Figure 3:**
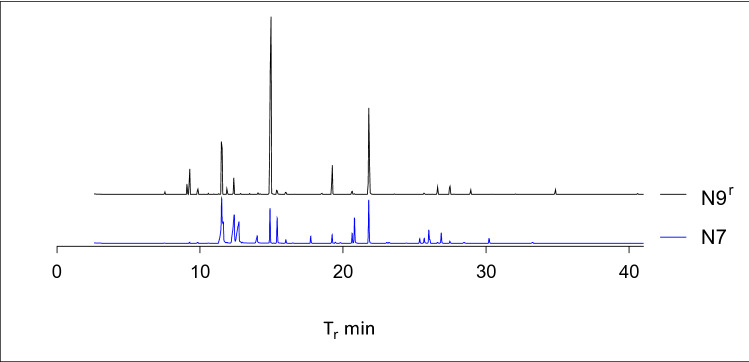
Sample comparison of two neroli samples as seen on GC-FID.

By comparing for example N7 and N9^r^ (Fig. 3), broad peaks in N7 are clearly visible and represent dipropylene glycol adulteration. There is a marked difference in linalool concentration at 15 min between the two samples. N7 also has significantly higher concentration of 2-phenylethanol, a naturally occurring compound in neroli found in the reference at about 1.1% but 5.24% in N7.

N10, although considered reference for its olfactory quality, does not met all specifications.

### Analysis of mandarin essential oil (MEO)

Mandarin oil is particular in the industry as the fruit is harvested in 3 stages of maturity; green, yellow and red mandarin oils can be sold as distinct oils, although the ISO norm only specifies Italian type mandarin. This range of harvest times affects the chemical composition of the oils but the three mandarin oils are not given individual ISO ranges. This increases the difficulty of determining if the variation of the composition is natural or the result of adulteration. Cold-pressed oils contain compounds too heavy or chemically unlikely to distill like waxes and furocoumarins. Citrus oils have characteristic furocoumarin compositions that can aid in the identification of adulteration. This approach was indeed successfully applied recently using either HPLC with columns with fused-core technology or UPLC-TOF–MS^40,41^.

Eleven commercial samples of MEO were analyzed and their peak areas were compared to ISO Norm 3528^42^. Two samples were acquired from reputable sources and considered premium reference samples. Of the eleven MEO samples, only one sample, M4, met all of the ISO chromatographic specifications. The ISO standard includes acceptable ranges for 7 compounds; α-pinene, β-pinene, myrcene, γ-terpinene, limonene, methyl-N-methylanthranilate, and α-sinensal.

The premium samples, M12 and M13 did not meet all 7 profiled compounds. M12 fell out of range for 4 compounds. M13 was high for β-pinene at 3.13 ± 0.19% rather than 2.0% max.

M1 and M2 were grossly out of specification in very similar fashion. Falling out of range for 6 compounds, only passing the myrcene specification. Each have about 95% limonene content which would suggest the oil is actually sweet orange essential oil. Methyl N-methylanthranilate was also not detected, an important odorant molecule for mandarin oil.

M3 poses an interesting question. Four of the regulated compounds are out of the ISO range, however, they are just slightly beyond the ranges. For example, ISO limits limonene to 65–75% area, this sample has 76.15 ± 0.04%; similarly, γ-terpinene is set from 16 to 22% with M3 containing 15.35 ± 0.005%. The question then becomes, how hard are the limits of the ISO ranges? Is a sample really “bad” if it falls just beyond the prescribed ranges or should it be considered natural variability ?^43^ Besides the question of maturity of mandarins at the time of harvest mentioned above, linalool and linalyl acetate vary by several percent throughout a growing season of Egyptian neroli oil^44^, and α-terpineol, terpinen-4-ol, α- & β-phellandrene and camphene change within growing seasons of bergamot crops, with α-terpineol increasing consistently and dramatically throughout the season across different years^45^.

M4 and M10 came from the same company. M4 meets all requirements of the ISO mandarin reference. M10 was sold as tangerine and does not meet the requirements of the mandarin norm. There is no official ISO norm for tangerine but literature suggests that a limonene content greater than 90% and a γ-terpinene content near 4% are characteristic^46^. M10 has a limonene content of 93.4% and γ-terpinene at 2.9% seemingly in agreement with other tangerine oils.

M5, M8, and M9 are all very nearly within the specification of the ISO standard. M5 met all specified ranges excluding α-sinensal. M8 is just below the range for methyl N-methylanthranilate and α-sinensal was not identified, with all other compounds within the ranges. M9 had a limonene content of 76.12 ± 0.03% and a γ-terpinene content of 15.64 ± 0.01%. These minor deviations from the ISO profile could be considered natural variations within an oil.

M11 only met the specification for limonene, the other 6 compounds falling below the required ranges or not identified at all. Uncharacteristically, this oil also contained linalool and linalyl acetate at over 10% each. This could be a sign of adulteration, potentially to improve the scent.

Furocoumarins contents were evaluated for the whole set of mandarin samples by reversed-phase HPLC–PDA analysis using 5-methylpsoralen^47^ as analytical reference. M1, M2 and M10 showed the same furocoumarin pattern as sweet orange oil taken as reference. Most samples did not undergo furocoumarin removal process. Orange oil being sold with a label of mandarin oil can more than double the sale price (from $0.20 to $0.57 per mL from one source) to consumers that may not have a nose sensitive enough to smell the differences.

### Analysis of bergamot essential oil (BEO)

Eleven commercial samples of bergamot essential oil were analyzed and their peak areas were compared to ISO Norm 3520^48^. Two samples were acquired from reputable sources and considered premium reference samples. Of the eleven BEO samples, only one sample, B6, met all of the ISO chromatographic specifications. The ISO standard includes acceptable ranges for 7 compounds: β-pinene, γ-terpinene, limonene, linalool, linalyl acetate, geranial, and β-bisabolene.

B1 and B2, purchased at 12.5 cents/mL, were subject to gross dilution. B1 had a large peak identified as triethyl citrate (TEC) comprising 60% of the total peak area. Excluding the TEC peak, B1 meets the relative peak area requirements of the ISO standard. B2 also meets the peak area requirements of the ISO standard, but is heavily diluted with a vegetal oil that is not visible by GC directly. These oils are likely genuine bergamot essential oils that have been diluted with a known perfumery solvent. ^1^H-NMR analysis indeed showed in B1 characteristic signals of TEC with 2 quadruplets at 4.8–4.0 ppm, 2 doublets with strong coupling constants and roof effect at 2.9–2.5 ppm, and 2 triplets at 1.3–1.2 ppm. In B2, a pattern consisting of ethylenic, allylic and aliphatic signals characteristic of soybean oil was observed (see S.I.).

The majority of the bergamot samples nearly met all of the ISO prescription except for either the geranial or β-bisabolene ranges. B3 was just over the range for β-pinene at 9.58 ± 0.00% and below the 0.3% minimum β-bisabolene level, with all other compounds within the range. B4 and B10 also fell out of range for β-bisabolene. B4, B11 and B12, were lower than the 0.25% minimum geranial specification, B4 being nearly at the limit with 0.24 ± 0.004%. B10 has a second compound out of range, γ-terpinene, which is 5.83 ± 0.02%, below the 6% AFNOR value. B9 falls just out of range for β-pinene at 5.43 ± 0.38%, but this standard deviation would suggest the β-pinene can be considered within the range. B7 was in range for all compounds except geranial. B5 has 3 compounds out of range, but to a very small extent. These samples are all very close to being acceptable according to the AFNOR ranges. They are unlikely to be adulterated, but likely graded as lower odor quality by the perfume industry and sold to the essential oil industry.

B6 is entirely conformant to the ISO reference. B7, purchased from a French pharmacy, was fully compliant except for geranial again, possibly due to aging, aldehydes such as geranial and neral being lost upon oxidation to the corresponding carboxylic acids or cyclized and dehydrated to *para*-cymene^49,50^.

Reverse-phase HPLC–PDA analysis of bergamot samples revealed the presence of 5-methylpsoralen and a closely related peak, and at significantly lower concentrations in highly diluted samples B1 and B2. Furocoumarin free (FCF) samples; B4, B10, B15, and B16 contained neither of these peaks, but had defaults in their composition in geranial and β-bisabolene.

### Graphical representation with heat maps

Statistical manipulation follows a modified Z-score analysis. Standard Z-scores are calculated using the statistical mean and standard deviation.

$$z=\frac{x-\mu }{\sigma }$$

The score relays the relative distance of a data point to the mean, a Z-score less than one would place the data point within the first standard deviation of the data set.

In this analysis, the Z-score calculation was modified to reflect the AFNOR/ISO guidelines. The mid-point of the range replaces the statistical average (µ), and the distance between the mid-point and the edge of the range becomes the standard deviation ($\sigma )$. This results in a score of the distance of each compound from the accepted value, normalized to the magnitude of the range. This allows for easier comparison of compounds present at varying levels and with different specifications.

**Figure 4:**
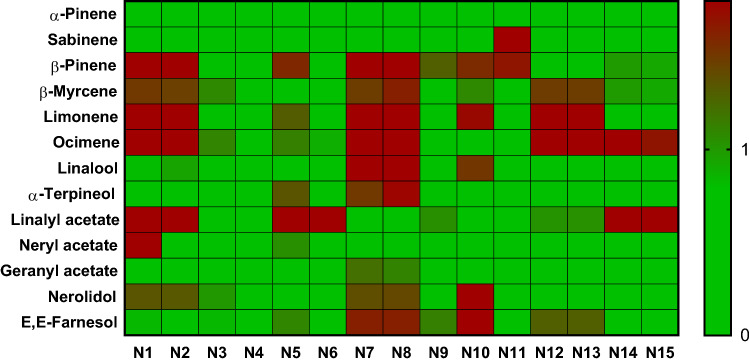
Heat map of relative scores of neroli essential oils for relevant compounds according to ISO 3517. N9 and N10 were obtain from a reputable source.

**Figure 5:**
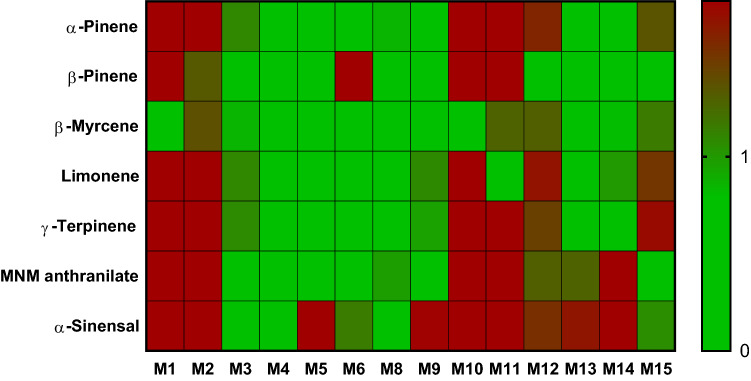
Heat map of relative scores of mandarin essential oils for relevant compounds according to ISO 3528. M12 and M13 were obtain from reputable source.

**Figure 6:**
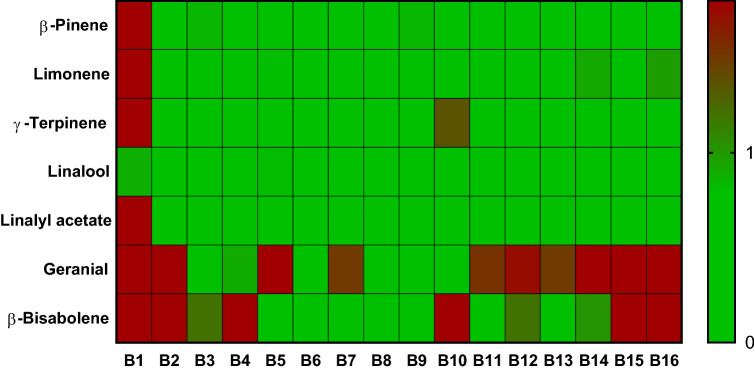
Heat map of relative scores of bergamot essential oils for relevant compounds according to ISO 3520. B11 and B12 were obtained from reputable sources.

Heat maps were generated by applying the modified Z-score calculation to each sample’s compounds of interest per the AFNOR standard to which were added 9 additional samples. A score less than or equal to one is green, signifying the acceptable level of the compound. Scores over 1 are (gradient from green to red) with the intensity reflecting the severity of the distance from the accepted value. This visual representation shows quickly and intuitively if a sample is compliant or far from the AFNOR guidelines (Figs. 4, 5, 6).

### Price/quality relationship

Price per milliliter of oil was calculated based on the purchase price in USD, excluding tax, and the container volume per label. Samples were chosen from online retailers with the intention of obtaining a range of price points. The hypothesis being that adulteration would be more prevalent in samples with a lower price/mL. Economically motivated adulteration is the unethical practice of adding material to a product to increase profits. To add diluent to an oil sold well below the market value would allow the seller to make a profit even at a reduced price, a buyer could predict that the quality of the product may be compromised when buying at a deep discount. To add diluent to an oil sold at or above market value would significantly increase the profits of the seller while falsely assuring the buyer that the oil is of the quality expected at the higher price point.

**Figure 7:**
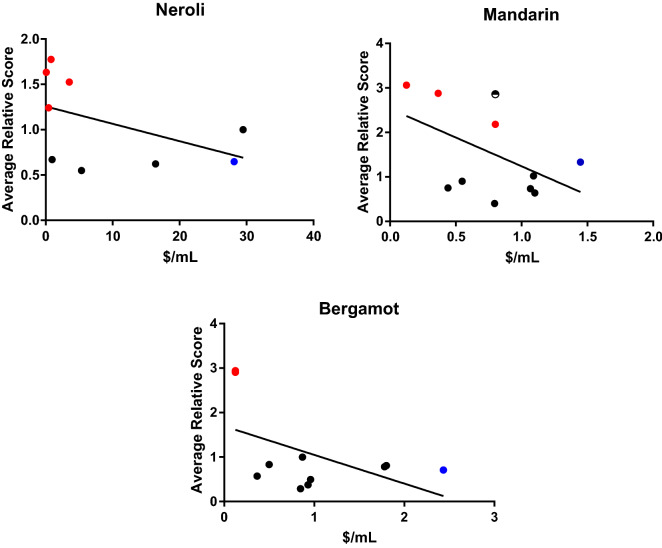
The average relative score of each sample is plotted against the price in USD per milliliter of essential oil (red: adulterated; black: acceptable; blue: reference; half filled circle is the tangerine sample).

Because of neroli’s high price, it is of particular interest in this study. Neroli is the steam distilled oil of bitter orange flowers, floral EOs are often expensive due to the delicate harvest procedures and low oil yields^51^. Additionally, because neroli blossoms become oranges if left to be pollinated on the tree, there is often a limit on neroli harvest in order to not impact the later bitter orange harvest. The price per milliliter of neroli in our sample set ranged from $0.17/mL to $29.50/mL. Sample N4, the only sample to meet all of the ISO guidelines for neroli EO was purchased at $5.31/mL, falling about the median price of our sample list while the most expensive sample, N5, failed to meet 6 out of 13 of the requisite values (Fig. 7).

Mandarin is a considerably less expensive oil. The price range of the samples analyzed here was $0.12-$1.45/mL, with the average price/mL equal to $0.78.

This data does not give a clear prescriptive for purchasing a quality essential oil. The problem is not resolved by simply buying the most expensive oil. One could imagine that advice being quite dangerous to the market, flooding it with overpriced low-quality oils. The transparency and the quality control standards of businesses then become the consumers’ guides for purchasing.

## Conclusion

Regulatory bodies will struggle to police the online world of consumer products because of the magnitude of the industry. It would be impossible to test every essential oil in the online market the way we have for these 31 products. Policing the market becomes even more difficult when a company that exists only online can change names or raw materials with little to no traceability. Label control seems non-existent with nearly all labels including some version of the words 100% pure, natural, or therapeutic grade essential oil. Most labels include verbiage to dilute essential oils as they are highly concentrated and can irritate skin, even on samples that had been heavily diluted already. There is also the question of who is at fault when an oil is sold adulterated. Is the adulteration happening at the raw material stage and the seller is only guilty of not verifying their raw materials? Or are the sellers buying quality raw materials and adulterating the products themselves? The surest choice for one consumer at the end of this complex value chain seems to be to purchase essential oils from known suppliers with a brand strategy, generally running rigorous quality control.

## Materials and methods

Commercial EO samples were obtained through internet retailers and French pharmacies. Premium samples were obtained from reputable fragrance houses. Nine (9) bergamot (*Citrus bergamia*) essential oil samples were purchased from online retailers, and 1 was purchased in a French pharmacy. Eight (8) mandarin (*Citrus reticulata*) essential oil samples were purchased online, 1 from a French pharmacy. One (1) Tangerine sample was also purchased online to compare to the mandarin samples. Eight (8) neroli (*Citrus aurantium*) essential oil samples were purchased from online retailers.

An Agilent Technologies gas chromatograph (6890) was used with a flame ionization detector (FID), and an Agilent J&W HP-5 column with dimensions 30 m × 0.32 mm ID, with 0.25 µm film thickness. 1 μL of diluted essential oil (20 μL sample and 20 μL of 5 mg/mL methyl octanoate internal standard diluted to 1.0 mL with ethyl acetate) was injected in split mode at 10:1. Oven temperature was programmed at 50 °C for 3 min, then to 265 °C at 3 °C/min, a final ramp to 300 °C at 15 C/min and 5 min hold. Injector and detector temperatures were set at 265 °C. Hydrogen was the carrier gas at a constant flow of 1 mL/min. Linear retention indices were calculated with reference to n-alkanes (C7-C30).

GC–MS analyses were performed on a 7890A GC system coupled to a 5975C VL mass spectrometer detector (Agilent Technologies) equipped with an Agilent J&W HP-5 column with dimensions 30 m × 0.32 mm ID, with 0.25 µm film thickness. 1 μL of diluted essential oil (20 μL sample and 20 μL of 5 mg/mL methyl octanoate internal standard diluted to 1.0 mL with ethyl acetate) was injected in split mode at 10:1. The GC–MS experimental conditions developed in the laboratory were the same as GC-FID analysis except for injector and detector temperatures (200 °C); carrier gas (helium); ionization voltage 70 eV; electron multiplier, 1 kV. Compound identification was accomplished through comparison of their mass spectra to NIST05 libraries as well as by comparison of their retention indices literature data. EOs compositions are given as relative area percentages. See SI for more details.

## Supplementary Information

Supplementary Information.

## References

[1] Rubiolo P, Sgorbini B, Liberto E, Cordero C, Bicchi C (2010). Essential oils and volatiles: Sample preparation and analysis. A review. Flavour Fragr. J..

[2] Bicchi C, Chaintreau A, Joulain D (2018). Identification of flavour and fragrance constituents. Flavour Fragr. J..

[3] Cachet T (2016). IOFI recommended practice for the use of predicted relative-response factors for the rapid quantification of volatile flavouring compounds by GC-FID. Flavour Fragr. J..

[4] Dhami N, Mishra AD (2015). Phytochemical variation: How to resolve the quality controversies of herbal medicinal products?. J. Herbal Med..

[5] Baser KHC, Buchbauer G (2015). Handbook of Essential Oils: Science, Technology, and Applications.

[6] Puškárová A, Bučková M, Kraková L, Pangallo D, Kozics K (2017). The antibacterial and antifungal activity of six essential oils and their cyto/genotoxicity to human HEL 12469 cells. Sci. Rep..

[7] Sipe HJ, Lardinois OM, Mason RP (2014). Free radical metabolism of methyleugenol and related compounds. Chem. Res. Toxicol..

[8] Auerbach SS (2010). Predicting the hepatocarcinogenic potential of alkenylbenzene flavoring agents using toxicogenomics and machine learning. Toxicol. Appl. Pharmacol..

[9] Rietjens IMCM (2005). Flavonoids and alkenylbenzenes: Mechanisms of mutagenic action and carcinogenic risk. Mutat. Res.-Fund. Mol. Mech. Mutagen..

[10] Abdo KM (2001). 14-week toxicity and cell proliferation of methyleugenol administered by gavage to F344 rats and B6C3F1 mice. Food Chem. Toxicol..

[11] Johnson JD (2000). Two-year toxicity and carcinogenicity study of methyleugenol in F344/N rats and B6C3F1 mice. J. Agric. Food Chem..

[12] Burkey JL, Sauer J-M, McQueen CA, Glenn Sipes I (2000). Cytotoxicity and genotoxicity of methyleugenol and related congeners—A mechanism of activation for methyleugenol. Mutat. Res.-Fund. Mol. Mech Mutagen..

[13] Chan VSW, Caldwell J (1992). Comparative induction of unscheduled DNA synthesis in cultured rat hepatocytes by allylbenzenes and their 1′-hydroxy metabolites. Food Chem. Toxicol..

[14] Melough MM, Cho E, Chun OK (2018). Furocoumarins: A review of biochemical activities, dietary sources and intake, and potential health risks. Food Chem. Toxicol..

[15] Sage E (1989). Oxidative DNA damage photo-induced by 3-carbethoxypsoralen and other furocoumarins: Mechanisms of photo-oxidation and recognition by repair enzymes. J. Mol. Biol..

[16] Sarkic A, Stappen I (2018). Essential oils and their single compounds in cosmetics—A critical review. Cosmetics.

[17] Asztemborska, M. & Ochocka, J. R. In *Studies in Natural Products Chemistry* Vol. 27 (ed Rahman, A.) 361–391 (Elsevier, 2002).

[18] Delort E (2015). Comparative analysis of three Australian finger lime (*Citrus australasica*) cultivars: Identification of unique citrus chemotypes and new volatile molecules. Phytochemistry.

[19] Adorjan B, Buchbauer G (2010). Biological properties of essential oils: An updated review. Flavour Fragr. J..

[20] Dobetsberger C, Buchbauer G (2011). Actions of essential oils on the central nervous system: An updated review. Flavour Fragr. J..

[21] E.M. Mustafa N (2015). Citrus essential oils: Current and prospective uses in the food industry. Recent Pat. Food Nutr. Agric..

[22] Hąc-Wydro K, Flasiński M, Romańczuk K (2017). Essential oils as food eco-preservatives: Model system studies on the effect of temperature on limonene antibacterial activity. Food Chem..

[23] Mahato N (2019). Citrus essential oils: Extraction, authentication and application in food preservation. Crit. Rev. Food Sci. Nutr..

[24] de Groot A (2019). Limonene hydroperoxides. Dermatitis.

[25] European Commission. Scientific Committee on Consumer Products (SCCP). Opinion on Furocoumarins in Cosmetic Products. Report No. SCCP/0942/0905 (2005), https://ec.europa.eu/health/ph_risk/committees/04_sccp/docs/sccp_o_036.pdf, Accessed 27 Apr 2021.

[26] Swift KAD (2004). Catalytic transformations of the major terpene feedstocks. Top. Catal..

[27] Do TKT, Hadji-Minaglou F, Antoniotti S, Fernandez X (2015). Essential oil authenticity: A challenge for the analytical chemist. Trends Anal Chem..

[28] ISO, *Essential oils*. In Standards catalogue, ICS 71.100.60.

[29] T. K. T. Do, S. Antoniotti, X. Fernandez. Essential oil authenticity. *Trends Anal. Chem.* **66**, 146–157 (2015).

[30] Gershon H, Lykkeberg A, Goren F, Mason S (2019). Identifying fraudulent natural products: A perspective on the application of carbon-14 analysis. J. Agric. Food Chem..

[31] Cagliero C (2016). Enantioselective gas chromatography with derivatized cyclodextrins in the flavour and fragrance field. Isr. J. Chem..

[32] Lebanov L, Tedone L, Kaykhaii M, Linford MR, Paull B (2019). Multidimensional gas chromatography in essential oil analysis. Part 2: Application to characterisation and identification. Chromatographia.

[33] Cerceau CI, Barbosa LCA, Alvarenga ES, Maltha CRA, Ismail FMD (2020). ^1^H-NMR and GC for detection of adulteration in commercial essential oils of *Cymbopogon* ssp. Phytochem. Anal..

[34] Mbogning Feudjio W (2017). Fluorescence spectroscopy combined with chemometrics for the investigation of the adulteration of essential oils. Food Anal. Methods.

[35] Bounaas K (2018). Essential oil counterfeit identification through middle infrared spectroscopy. Microchem. J..

[36] VargasJentzsch P, Gualpa F, Ramos LA, Ciobotă V (2018). Adulteration of clove essential oil: Detection using a handheld Raman spectrometer. Flavour Fragr. J..

[37] Lee KA, Harnett JE, Cairns R (2020). Essential oil exposures in Australia: Analysis of cases reported to the NSW Poisons Information Centre. Med. J. Aust..

[38] https://www.iso.org/fr/standard/45645.html, Accessed 17 Apr 2020.

[39] West TFC (1948). Synthetic perfumes. Sci. Prog..

[40] Russo M (2015). Reduced time HPLC analyses for fast quality control of citrus essential oils. J. Essent. Oil Res..

[41] Masson J (2016). Oxygenated heterocyclic compounds to differentiate *Citrus* spp. essential oils through metabolomic strategies. Food Chem..

[42] https://www.iso.org/fr/standard/53970.html, Accessed 17 Apr 2020.

[43] König WA, Fricke C, Saritas Y, Momeni B, Hohenfeld G (1997). Adulteration or natural variability? Enantioselective gas chromatography in purity control of essential oils. J. High Resolut. Chromatogr..

[44] Bonaccorsi I (2011). Composition of Egyptian nerolì oil. Nat. Prod. Commun..

[45] Dugo G (2012). Characterization of cold-pressed and processed bergamot oils by using GC-FID, GC-MS, GC-C-IRMS, enantio-GC, MDGC, HPLC and HPLC-MS-IT-TOF. J. Essent. Oil Res..

[46] Reeve D, Treatt R, Arthur D, Treatt F (2002). Riding the citrus trail: When is a mandarin a tangerine ?. Perfumer Flavorist.

[47] Dugrand-Judek A (2015). The distribution of coumarins and furanocoumarins in citrus species closely matches citrus phylogeny and reflects the organization of biosynthetic pathways. PLoS ONE.

[48] https://www.iso.org/fr/standard/8893.html, Accessed 17 Apr 2020.

[49] Turek C, Stintzing FC (2013). Stability of essential oils: A review. Compr. Rev. Food Sci. Food Saf..

[50] Jakab E (2018). Thermo-oxidative decomposition of lime, bergamot and cardamom essential oils. J. Anal. Appl. Pyrolysis.

[51] Sarrou E, Chatzopoulou P, Dimassi-Theriou K, Therios I (2013). Volatile constituents and antioxidant activity of peel, flowers and leaf oils of *Citrus aurantium* L. growing in Greece. Molecules.

