# Supplementary material for: Type and magnitude of non-compliance and adulteration in neroli, mandarin and bergamot essential oils purchased on-line: potential consumer vulnerability
Source: Sci Rep. 2021 May 27;11:11096. doi: 10.1038/s41598-021-90307-2 (PMC8160360; doi:10.1038/s41598-021-90307-2)
Supplement: Supplementary file 1 — Supplementary Information. [file 41598_2021_90307_MOESM1_ESM.docx]

Type and magnitude of non-compliance and adulteration in neroli, mandarin and bergamot essential oils purchased on-line: potential consumer vulnerability

Marissa Pierson,^1,2^ Xavier Fernandez,^2^ Sylvain Antoniotti^2,3^*

*^1^ European Research Institute on Natural Ingredients (ERINI), 4 Traverse Dupont, 06130 Grasse, FRANCE.*

*^2^ Université Cote d’Azur, CNRS, Institut de Chimie de Nice, Parc Valrose, 06108 Nice Cedex 2, FRANCE.*

*^3^ Université Cote d’Azur, Institute for Innovation and Partnerships in Flavor, Fragrance, Cosmetics, Grasse Biotech, 45 Bd Marcel Pagnol, 06130 Grasse, FRANCE.*

Correspondence: [sylvain.antoniotti@univ-cotedazur.fr](mailto:sylvain.antoniotti@univ-cotedazur.fr)

**Outline**

1. **Sampling S2**
2. **GC analyses S2**
3. **NMR spectroscopy S14**
4. **Graphical representation with heat Maps S15**
5. **HPLC analysis S16**
6. **Sampling**

Commercial EO samples were obtained through internet retailers and French pharmacies. Premium samples were obtained from reputable fragrance houses. With 23 samples out of ca. 230 made available from a large platform operating on-line, the sampling size was deemed sufficient to have a first estimate (±20% at the 95% level of confidence, for a normal distribution). To these 23 samples were added 8 additional samples purchased from other retail sources, mostly online as well.

Nine (9) bergamot (*Citrus bergamia*) essential oil samples were purchased from online retailers, and 1 was purchased in a French pharmacy. Eight (8) mandarin (*Citrus reticulata*) essential oil samples were purchased online, 1 from a French pharmacy. One (1) Tangerine sample was also purchased online to compare to the mandarin samples. Eight (8) neroli (*Citrus aurantium*) essential oil samples were purchased from online retailers.

1. **GC analyses**

An Agilent Technologies gas chromatograph (6890) was used with a flame ionization detector (FID), and an Agilent J&W HP-5 column with dimensions 30m x 0.32mm ID, with 0.25µm film thickness. 1 μL of diluted essential oil (20 μL sample and 20 μL of 5mg/mL methyl octanoate internal standard diluted to 1.0 mL with ethyl acetate) was injected in split mode at 10:1. Oven temperature was programmed at 50 C for 3 minutes, then to 265 C at 3 C/min, a final ramp to 300 C at 15 C/min and 5 min hold. Injector and detector temperatures were set at 265 °C. Hydrogen was the carrier gas at a constant flow of 35 mL/min. Linear retention indices were calculated with reference to n-alkanes (C7-C30).

GC–MS analyses were performed on a 7890A GC system coupled to a 5975C VL mass spectrometer detector (Agilent Technologies) equipped with an Agilent J&W HP-5 column with dimensions 30m x 0.32mm ID, with 0.25µm film thickness. 1 μL of diluted essential oil (20 μL sample and 20 μL of 5mg/mL methyl octanoate internal standard diluted to 1.0 mL with ethyl acetate) was injected in split mode at 10:1. The GC-MS experimental conditions developed in the laboratory were the same as GC-FID analysis except for injector and detector temperatures (200 °C); carrier gas (helium); ionization voltage 70 eV; electron multiplier, 1 kV. Besides main compounds used in ISO standards that have been authenticated by comparison with authentic samples (α-pinene, limonene, linalool, linalyl acetate, nerolidol, farnesol), compounds identification was accomplished through comparison of their mass spectra to NIST05 libraries as well as by comparison of their retention indices literature data using AMDIS deconvolution software.

EOs compositions are given as relative area percentages calculated from triplicate analysis. Peak areas are adjusted for the internal standard by subtracting the internal peak area from the total peak area before calculating percent areas: Area%_peak n_=((100*A_peak n_)/(A_total­_­ – A_ISTD_)).

|  |  | **Table 1:** Neroli peak areas for samples with typical profiles | | | | | | | | | | | | | | |
| --- | --- | --- | --- | --- | --- | --- | --- | --- | --- | --- | --- | --- | --- | --- | --- | --- |
| **#** | **RI** | **RI lit** | **Compound** | **ISO** | **N3** | **N4** | **N5** | **N6** | **N9^r^** | **N10^r^** | **N11** | **N12** | **N13** | **N14** | **N15** |  |
|  |  |  |  | **ranges** | % | % | % | % | % | % | % | % | % | % | % |  |
| 1 | 923 | 923 | α-thujene |  | TR | TR | TR | TR | TR | -- | 0.11 | TR | TR | TR | TR |  |
| 2 | 930 | 937 | α -pinene | 0-2 | 1.06 | 1.76 | 0.29 | 0.52 | 0.40 | 'TR | 0.64 | 0.58 | 0.57 | 0.37 | 0.41 |  |
| 3 | 970 | 975 | sabinene | 0-3 | -- | -- | 0.76 | -- | 1.27 | 0.50 | 7.23 | -- | -- | -- | -- |  |
| 4 | 974 | 975 | β-pinene | 7-17 | 14.15 | 15.13 | 3.86 | 8.77 | 5.03 | 4.08 | 3.41 | 8.80 | 8.65 | 6.59 | -- |  |
| 5 | 988 | 989 | β-myrcene | 1-4 | 0.67 | 1.29 | 2.30 | 1.37 | 1.67 | 0.66 | 1.87 | 0.20 | 0.21 | 0.79 | 0.94 |  |
| 6 | 1006 | 995 | carene |  | 0.82 | TR | TR | TR | 0.14 | TR | 0.58 | TR | TR | -- | -- |  |
| 7 | 1008 | 1018 | α-terpinene |  | -- | TR | TR | -- | TR | TR | 0.21 | -- | -- | -- | -- |  |
| 8 | 1022 | 1026 | paracymene |  | 0.81 | TR | 0.31 | 0.20 | TR | TR | 0.68 | 0.25 | 0.24 | 0.10 | 0.11 |  |
| 9 | 1027 | 1029 | limonene | 9-18 | 13.38 | 15.86 | 7.16 | 9.93 | 13.61 | 5.59 | 9.21 | 5.06 | 4.98 | 9.34 | 10.77 |  |
| 10 | 1035 | 1049 | (*Z*)-ocimene | 3-8 | 2.01 | 1.20 | 0.49 | 0.32 | 0.89 | 0.36 | 0.47 | TR | TR | 0.12 | 0.16 |  |
| 11 | 1046 | 1051 | (*E*)-ocimene |  | TR | 3.23 | 1.86 | 2.54 | 2.40 | 4.26 | 4.26 | TR | TR | 0.80 | 1.15 |  |
| 12 | 1056 | 1062 | γ-terpinene |  | 0.42 | TR | TR | TR | 0.13 | 0.22 | 0.55 | TR | TR | -- | -- |  |
| 13 | 1070 | 1070 | linalool oxide* |  | 0.19 | TR | 0.48 | 0.15 | 0.21 | TR | 0.15 | -- | -- | -- | -- |  |
| 14 | 1083 | 1088 | terpinolene |  | 0.87 | 0.52 | 0.21 | 0.12 | 0.24 | 0.30 | 0.44 | 1.90 | 1.89 | 0.49 | 0.48 |  |
| 15 | 1086 | 1078 | linalool oxide* |  | -- | -- | 0.23 | 0.12 | 0.23 | TR | 0.13 | 1.69 | 1.68 | 0.41 | 0.40 |  |
| 16 | 1103 | 1100 | linalool | 28-44 | 36.49 | 38.35 | 39.64 | 33.74 | 42.13 | 48.42 | 40.57 | 42.42 | 42.23 | 37.53 | 39.52 |  |
| 17 | 1112 | 1114 | 2-phenylethanol |  | -- | 0.35 | 0.56 | TR | 1.11 | TR | TR | -- | -- | TR | TR |  |
| 18 | 1125 | 1127 | methyl octanoate^i^ |  | 0.41 | 0.41 | 0.47 | 0.47 | 0.45 | 0.43 | 0.42 | 0.57 | 0.56 | 0.50 | 0.53 |  |
| 19 | 1169 | 1173 | epoxylinalol |  | -- | TR | TR | 0.11 | TR | TR | TR | 0.89 | 0.89 | 0.16 | 0.15 |  |
| 20 | 1178 | 1187 | terpinen-4-ol* |  | 0.75 | 0.10 | 0.37 | 0.26 | 0.29 | 0.86 | 1.28 | 0.49 | 0.49 | 0.35 | 0.34 |  |
| 21 | 1186 | 1181 | paracymen-8-ol* |  | TR | -- | -- | TR | -- | -- | TR | 0.16 | 0.15 | TR | TR |  |
| 22 | 1194 | 1191 | α−terpineol | 2-5.5 | 2.99 | 3.52 | 6.30 | 3.16 | 4.64 | 4.67 | 3.85 | 4.60 | 4.60 | 4.11 | 4.34 |  |
| 23 | 1191 | 1186 | (*E*)-3-hexenyl butyrate* |  | -- | TR | 0.14 | 0.11 | TR | TR | TR | 2.03 | 1.79 | 0.19 | 0.16 |  |
| 24 | 1199 | 1199 | γ-terpineol |  | 0.56 | -- | -- | -- | -- | -- | -- | -- | 0.69 | -- | -- |  |
| 25 | 1224 | 1255 | geraniol |  | -- | 1.22 | 1.85 | 0.82 | 1.35 | 1.16 | 0.96 | 0.57 | 0.57 | 0.83 | 0.88 |  |
| 26 | 1228 | 1232 | citronellol* |  | 2.76 | -- | -- | -- | -- | -- | -- | TR | TR | TR | TR |  |
| 27 | 1242 | 1242 | carvone* |  | TR | -- | TR | TR | TR | -- | TR | 0.25 | 0.25 | TR | TR |  |
| 28 | 1251 | 1253 | linalyl acetate | 3-15 | 12.33 | 8.27 | 20.44 | 21.51 | 16.26 | 5.31 | 9.87 | 15.96 | 15.89 | 25.39 | 26.75 |  |
| 29 | 1269 | 1256 | hydroxycitronellol |  | 0.21 | -- | 0.14 | 0.11 | TR | -- | TR | 0.14 | 0.14 | -- | -- |  |
| 30 | 1274 |  | β-terpenyl acetate |  | -- | -- | -- | -- | -- | -- | TR | 0.33 | 0.33 | TR | TR |  |
| 31 | 1275 | 1282 | geranyl formate* |  | -- | -- | TR | TR | -- | -- | TR | 0.23 | 0.24 | TR | TR |  |
| 32 | 1283 | 1289 | indole |  | -- | -- | TR | -- | -- | TR | -- | 0.25 | 0.25 | TR | TR |  |
| 33 | 1298 | 1292 | lavandulyl acetate* |  | -- | -- | 0.14 | TR | -- | -- | TR | 0.72 | 0.72 | 0.26 | 0.26 |  |
| 34 | 1332 | 1341 | methyl anthranilate |  | -- | 0.39 | 0.35 | -- | 0.34 | 0.23 | -- | -- | -- | 0.36 | 0.39 |  |
| 35 | 1334 |  | sabinyl acetate* |  | TR | -- | -- | TR | -- | -- | -- | 0.55 | 0.56 | -- | -- |  |
| 36 | 1344 | 1275* | terpenyl acetate |  | 0.56 | TR | 0.19 | 0.12 | TR | 0.28 | TR | 0.23 | 0.23 | 0.15 | 0.14 |  |
| 37 | 1359 | 1362 | neryl acetate | 0-2.5 | 0.10 | 1.42 | 2.78 | 1.68 | 1.26 | 1.77 | 1.60 | 1.43 | 1.43 | 1.37 | 1.41 |  |
| 38 | 1368 |  | α-copaene |  | TR | -- | TR | -- | -- | -- | TR | 0.30 | 0.30 | -- | -- |  |
| 39 | 1379 | 1383 | geranyl acetate | 1-5 | 1.84 | 2.44 | 4.72 | 3.13 | 2.03 | 3.42 | 3.05 | 2.56 | 2.55 | 2.51 | 2.63 |  |
| 40 | 1385 | 1369* | methyl N-methylanthranilate* |  | -- | TR | TR | TR | 0.13 | -- | 0.43 | TR | TR | -- | -- |  |
| 41 | 1413 |  | β-caryophyllene |  | 0.62 | 0.71 | 0.47 | 0.84 | 0.75 | 0.62 | 0.59 | TR | TR | 0.14 | 0.15 |  |
| 42 | 1560 | 1559 | nerolidol | 1-5 | 0.69 | 1.36 | 1.33 | 4.34 | 1.19 | 7.52 | 3.40 | 1.81 | 1.81 | 3.21 | 3.36 |  |
| 43 | 1576 |  | caryophyllene oxide isomer* |  | -- | TR | TR | 0.18 | TR | TR | 0.12 | 0.26 | 0.26 | 0.11 | 0.11 |  |
| 44 | 1648 |  | hedione isomer |  | 0.45 | -- | -- | TR | -- | TR | TR | -- | -- | -- | -- |  |
| 45 | 1691 | 1680 | farnesol isomer |  | 1.26 | -- | TR | 0.13 | TR | 0.14 | 0.21 | -- | -- | TR | TR |  |
| 46 | 1713 |  | *E*,*E-*farnesol | 1-4 | 0.97 | 1.30 | 0.65 | 2.53 | 0.63 | 6.64 | 1.70 | 0.42 | 0.42 | 1.54 | 1.64 |  |
|  |  | Notes: TR, Trace <0.1%; --, not identified; *Tentative identification (similarity <85%); ^i^Internal standard,  ^r^Reputable sample. | | | | | | | | | | | | | | |
|  |  | All results the average of three sample injections, % area excludes area of Methyl Octanoate ISTD. | | | | | | | | | | | | | | |

|  | |  | **Table 2:** Bergamot peak areas for samples with typical profiles | | | | | | | | | | | |  | |  | |  | |  |  |
| --- | --- | --- | --- | --- | --- | --- | --- | --- | --- | --- | --- | --- | --- | --- | --- | --- | --- | --- | --- | --- | --- | --- |
| **#** | **RI** | **RI lit** | **Compound** | **ISO** | **B3** | **B4** | **B5** | **B6** | **B7** | **B8** | **B9** | **B10** | **B11^r^** | **B12^r^** | | **B13** | | **B14** | | **B15** | | **B16** |
|  |  |  |  | **ranges** | % | % | % | % | % | % | % | % | % | % | | % | | % | | % | | % |
| 1 | 923 | 923 | α-thujene |  | 0.22 | 0.21 | 0.22 | 0.23 | 0.27 | 0.23 | 0.25 | 0.16 | 0.31 | 0.19 | | 0.18 | | 0.22 | | 0.20 | | 0.18 |
| 2 | 930 | 937 | α-pinene |  | 1.35 | 1.13 | 1.22 | 1.27 | 1.72 | 1.25 | 0.98 | 1.27 | 1.20 | 1.15 | | 1.07 | | 1.21 | | 1.16 | | 1.06 |
| 3 | 970 | 975 | sabinene |  | -- | -- | -- | -- | -- | -- | -- | 0.96 | -- | -- | | -- | | -- | | -- | | -- |
| 4 | 974 | 975 | β-pinene | 5.5-9.5 | 9.68 | 7.74 | 7.75 | 7.75 | 7.92 | 7.74 | 5.72 | 5.88 | 8.18 | 7.45 | | 6.72 | | 7.52 | | 6.99 | | 6.86 |
| 5 | 988 | 989 | β-myrcene |  | 1.00 | 1.10 | 0.94 | 1.27 | 1.01 | 1.27 | 1.11 | 0.93 | 1.04 | 1.11 | | 1.07 | | 1.15 | | 1.11 | | 1.13 |
| 6 | 1008 | 1018 | α-terpinene |  | TR | 0.12 | 0.21 | 0.16 | 0.10 | 0.15 | TR | TR | 0.13 | TR | | TR | | TR | | TR | | TR |
| 7 | 1027 | 1029 | limonene | 30-45 | 42.86 | 39.71 | 30.22 | 41.77 | 36.94 | 41.13 | 41.95 | 38.68 | 42.79 | 44.17 | | 42.07 | | 46.03 | | 44.65 | | 46.18 |
| 8 | 1046 | 1051 | ocimene |  | 0.11 | 0.21 | 0.18 | 0.24 | 0.25 | 0.23 | 0.23 | 0.13 | 0.18 | 0.15 | | 0.16 | | 0.17 | | 0.17 | | 0.18 |
| 9 | 1056 | 1062 | γ-terpinene | 6-10 | 6.15 | 6.07 | 6.28 | 7.17 | 6.21 | 7.22 | 6.18 | 5.13 | 6.10 | 6.50 | | 6.14 | | 7.02 | | 6.20 | | 6.56 |
| 10 | 1083 | 1088 | terpinolene |  | 0.19 | 0.25 | 0.47 | 0.32 | 0.26 | 0.29 | 0.21 | 0.24 | 0.31 | 0.19 | | 0.18 | | 0.20 | | 0.19 | | 0.21 |
| 11 | 1103 | 1100 | linalool | 3-15 | 8.64 | 12.76 | 15.30 | 8.32 | 12.33 | 8.82 | 14.44 | 13.48 | 11.81 | 9.64 | | 10.57 | | 8.39 | | 11.09 | | 10.74 |
| 12 | 1125 | 1127 | methyl octanoate^i^ |  | 0.36 | 0.31 | 0.38 | 0.32 | 0.39 | 0.37 | 0.34 | 0.38 | 0.38 | 0.35 | | 0.37 | | 0.39 | | 0.36 | | 0.33 |
| 13 | 1194 | 1191 | α-terpineol |  | 0.12 | TR | 0.19 | TR | 0.15 | TR | 0.14 | 0.20 | 0.11 | TR | | TR | | TR | | TR | | TR |
| 14 | 1211 |  | neral* |  | 0.23 | 0.12 | 0.37 | 0.19 | 0.12 | 0.19 | 0.17 | 0.21 | 0.11 | 0.10 | | TR | | TR | | 0.12 | | 0.11 |
| 15 | 1225 | 1253 | linalyl acetate | 22-36 | 27.43 | 28.63 | 33.27 | 28.55 | 30.46 | 28.69 | 26.22 | 30.34 | 25.31 | 27.43 | | 29.93 | | 25.98 | | 26.57 | | 25.40 |
| 16 | 1267 |  | geranial* | 0.25-0.5 | 0.40 | 0.25 | 0.64 | 0.30 | 0.18 | 0.29 | 0.26 | 0.30 | 0.18 | 0.16 | | 0.15 | | 0.14 | | 0.16 | | 0.12 |
| 17 | 1344 | 1275 | terpinyl acetate* |  | 0.11 | 0.17 | 0.15 | 0.15 | 0.14 | 0.15 | 0.10 | 0.23 | 0.18 | TR | | TR | | TR | | TR | | TR |
| 18 | 1359 | 1362 | neryl acetate |  | 0.33 | 0.39 | 0.44 | 0.50 | 0.42 | 0.51 | 0.38 | 0.52 | 0.42 | 0.34 | | 0.35 | | 0.36 | | 0.31 | | 0.28 |
| 19 | 1379 | 1383 | geranyl acetate |  | 0.28 | 0.34 | 0.41 | 0.44 | 0.28 | 0.45 | 0.28 | 0.41 | 0.35 | 0.34 | | 0.33 | | 0.42 | | 0.30 | | 0.28 |
| 20 | 1412 |  | β-caryophyllene |  | 0.16 | 0.23 | 0.33 | 0.34 | 0.32 | 0.34 | 0.36 | 0.27 | 0.36 | 0.24 | | 0.22 | | 0.24 | | 0.20 | | 0.18 |
| 21 | 1428 |  | bergamotene* |  | 0.15 | TR | 0.31 | 0.28 | 0.26 | 0.28 | 0.28 | 0.10 | 0.26 | 0.18 | | 0.17 | | 0.20 | | 0.13 | | 0.11 |
| 22 | 1504 |  | β-bisabolene | 0.3-0.55 | 0.26 | 0.11 | 0.55 | 0.41 | 0.38 | 0.41 | 0.42 | 0.13 | 0.38 | 0.26 | | 0.23 | | 0.28 | | 0.12 | | TR |
| 23 | 2048 |  | bergapten* |  | TR | TR | TR | TR | TR | -- | TR | TR | TR | -- | | -- | | -- | | -- | | -- |
|  | |  | Notes: TR, Trace <0.1%; --, not identified; *Tentative identification (similarity <85%); ^i^Internal standard, ^r^Reputable sample. All results the average of three sample injections, % area excludes area of Methyl Octanoate ISTD. | | | | | | | | | | | | | | | | | |  |  |
|  | |  |  | | | | | | | | | | | | | | | | | |  |  |

|  |  |  | **Table 3:** Mandarin peak areas for samples with typical profiles | | | | | | | | | | | |
| --- | --- | --- | --- | --- | --- | --- | --- | --- | --- | --- | --- | --- | --- | --- |
| **#** | **RI** | **RI Lit** | | **Compound** | **ISO** | **M3** | **M4** | **M5** | **M6** | **M8** | **M12^r^** | **M13^r^** | **M14** | **M15** |
|  |  |  | |  | **ranges** | % | % | % | % | % | % | % | % | % |
| 1 | 923 | 923 | | α-thujene |  | 0.50 | 0.77 | 0.75 | 0.67 | 0.68 | 0.52 | 0.44 | 0.44 | 0.61 |
| 2 | 930 | 937 | | α-pinene | 2-3 | 1.90 | 2.14 | 2.30 | 2.11 | 2.00 | 1.69 | 2.09 | 2.07 | 1.79 |
| 3 | 970 | 975 | | sabinene |  | 0.36 | -- | -- | -- | 0.21 | 0.18 | 0.24 | 0.23 | 0.24 |
| 4 | 974 | 975 | | β-pinene | 1.2-2 | 1.44 | 1.87 | 1.79 | 3.26 | 1.53 | 1.26 | 1.48 | 1.42 | 1.31 |
| 5 | 988 | 989 | | β-myrcene | 1.5-2 | 1.52 | 1.70 | 1.61 | 1.64 | 1.77 | 1.41 | 1.53 | 1.50 | 1.44 |
| 6 | 1022 | 1026 | | paracymene |  | 0.18 | 0.36 | 0.32 | 0.32 | 0.35 | 0.21 | 0.20 | 0.21 | 0.22 |
| 7 | 1027 | 1029 | | limonene | 65-75 | 76.87 | 71.51 | 74.29 | 72.59 | 74.91 | 79.03 | 74.71 | 76.19 | 78.54 |
| 8 | 1056 | 1062 | | γ-terpinene | 16-22 | 15.50 | 19.04 | 17.32 | 17.23 | 16.43 | 14.49 | 18.11 | 17.02 | 13.85 |
| 9 | 1083 | 1088 | | terpinolene |  | 0.55 | 0.84 | 0.75 | 0.69 | 0.77 | 0.60 | 0.48 | 0.45 | 0.61 |
| 10 | 1103 | 1100 | | linalool |  | 0.14 | 0.16 | 0.13 | 0.13 | 0.10 | TR | TR | TR | 0.18 |
| 11 | 1125 | 1127 | | methyl octanoate^i^ |  | 0.34 | 0.34 | 0.30 | 0.32 | 0.32 | 0.32 | 0.31 | 0.30 | 0.31 |
| 12 | 1178 | 1187 | | 1-terpinen-4-ol |  | TR | TR | TR | 0.19 | -- | TR | TR | TR | 0.12 |
| 13 | 1194 | 1191 | | α-terpineol |  | 0.17 | 0.23 | 0.11 | 0.27 | 0.29 | TR | 0.13 | TR | 0.21 |
| 14 | 1402 | 1369* | | Methyl N-methylanthranilate* | 0.3-0.6 | 0.38 | 0.53 | 0.35 | 0.49 | 0.29 | 0.24 | 0.24 | 0.14 | 0.34 |
| 15 | 1412 |  | | caryophyllene |  | TR | 0.15 | 0.12 | 0.10 | 0.13 | TR | TR | TR | 0.11 |
| 16 | 1503 |  | | α-farnesene |  | 0.15 | 0.38 | TR | 0.15 | 0.18 | TR | TR | TR | 0.25 |
| 17 | 1720 |  | | α-sinensal | 0.2-0.5 | 0.21 | 0.25 | TR | 0.16 | 0.29 | 0.11 | TR | TR | 0.17 |
|  | |  | Notes: TR, Trace <0.1%; --, not identified; *Tentative identification (similarity <85%); ^i^Internal standard, ^r^Reputable sample.  Al. All results the average of three sample injections, % area excludes area of Methyl Octanoate ISTD. | | | | | | | | | | | |
|  |  |  |  | | | | | | | | | | | |

|  | |  | **Table 4:** Neroli peak areas for samples with atypical profile | | | | |  |  |
| --- | --- | --- | --- | --- | --- | --- | --- | --- | --- |
| **#** | **RI** | **RI lit** | **Compound** | **ISO** | **N1** | **N2** | **N7** | **N8** |  |
|  |  |  |  | **ranges** | % | % | % | % |  |
| 1 | 770 |  | propylene glycol |  |  |  |  | 26.35 |  |
| 2 | 930 | 937 | α-pinene | 0-2 | TR | TR | TR |  |  |
| 3 | 970 | 975 | sabinene | 0-3 | -- | -- | TR |  |  |
| 4 | 974 | 975 | β-pinene | 7-17 | 0.94 | 0.87 | 0.73 |  |  |
| 5 | 988 | 989 | β-myrcene | 1-4 | 0.21 | 0.22 | 0.55 |  |  |
| 6 | 1006 | 995 | carene |  | 2.63 | 2.41 | TR |  |  |
| 7 | 1008 | 1018 | α-terpinene |  | -- | TR | -- |  |  |
| 8 | 1008-1150 |  | glycerol coelution |  | -- | -- | -- | 38.19 |  |
| 9 | 1022 | 1026 | paracymene |  | TR | TR | TR |  |  |
| 10 | 1027 | 1029 | limonene | 9-18 | 4.77 | 4.55 | 9.19 |  |  |
| 11 | 1045-1090 |  | DPG^ |  | 1.05 | 1.13 | 54.37 |  |  |
| 12 | 1056 | 1062 | γ-terpinene |  | -- | TR | -- |  |  |
| 13 | 1083 | 1088 | terpinolene |  | -- | TR | -- |  |  |
| 14 | 1086 |  | octyl formate* |  | -- | 0.49 | -- |  |  |
| 15 | 1103 | 1100 | linalool | 28-44 | 28.35 | 27.29 | 16.31 |  |  |
| 16 | 1112 | 1114 | 2-phenylethanol |  | TR | 0.13 | 9.75 |  |  |
| 17 | 1125 | 1127 | methyl octanoate^i^ |  | 1.34 | 0.52 | 1.31 |  |  |
| 18 | 1169 |  | benzyl acetate |  | -- | -- | 2.39 |  |  |
| 19 | 1194 | 1191 | α-terpineol | 2-5.5 | 4.09 | 4.06 | 2.97 | 0.21 |  |
| 20 | 1199 |  | γ-terpineol |  | 0.45 | 0.68 | 0.31 |  |  |
| 21 | 1216 |  | DPG* |  | 9.62 | 9.24 | -- |  |  |
| 22 | 1220 | 1232 | citronellol* |  | 1.00 | -- | -- |  |  |
| 23 | 1228 |  | citronellyl formate |  | -- | 1.08 | 16.36 |  |  |
| 24 | 1225 | 1253 | linalyl acetate | 3-15 | 27.30 | 26.67 | 8.93 | 1.39 |  |
| 25 | 1278 |  | β-terpenyl acetate* |  | -- | -- | 0.21 |  |  |
| 26 | 1290 | 1289 | indole* |  | 0.14 | TR | -- |  |  |
| 27 | 1300 | 1282 | geranyl formate* |  | -- | 0.13 | 0.25 |  |  |
| 28 | 1330 |  | 4-tert-butylcyclohexyl acetate* |  | -- | -- | 0.83 |  |  |
| 29 | 1337 |  | methyl anthranilate |  | 2.27 | 2.90 | 1.17 | 0.30 |  |
| 30 | 1344 | 1275* | terpenyl acetate |  | 5.62 | 5.51 | 3.26 |  |  |
| 31 | 1365 |  | 4-tert-butylcyclohexyl acetate* |  | -- | -- | 2.17 |  |  |
| 32 | 1359 | 1362 | neryl acetate | 0-2.5 | 0.72 | 0.75 | 0.55 |  |  |
| 33 | 1379 | 1383 | geranyl acetate | 1-5 | 4.41 | 4.40 | -- |  |  |
| 34 | 1402 | 1369* | methyl N-methylanthranilate* |  | 0.27 | 0.33 | 0.36 |  |  |
| 35 | 1444 |  | 2-methoxy-naphthalene, nerolin* |  | -- | -- | 0.92 |  |  |
| 36 | 1519 |  | nerolin new* |  | 0.49 | 0.69 | 0.22 |  |  |
| 37 | 1530 | 1559 | nerolidol | 1-5 | 0.12 | 0.11 | TR |  |  |
| 38 | 1560 |  | nerolidol isomer* |  | -- | 0.14 | TR |  |  |
| 39 | 1605 |  | methyl β-naphtyl ketone* |  | -- | 0.37 | TR |  |  |
| 40 | 1690 |  | (*E*,*E*)*-*farnesol | 1-4 | 1.45 | 1.51 | TR |  |  |
| 41 | 1713 | 1680 | farnesol isomer |  | 1.03 | 1.26 | TR |  |  |
|  |  |  |  |  |  |  |  |  |  |
|  | |  | Notes: TR, Trace <0.1%; --, not identified; *Tentative identification (similarity <85%); ^i^Internal standard.  All results the average of three sample injections, % area excludes area of Methyl Octanoate ISTD. | | | | | | |

|  | | |  | **Table 5:** Bergamot peak areas for samples with a-typical profiles | | | | |
| --- | --- | --- | --- | --- | --- | --- | --- | --- |
| **#** | **RI** | **RI Lit** | | | **Compound** | **ISO** | **B1** | **B2** |
|  |  |  | | |  | **ranges** | % | % |
| 1 | 923 | 923 | | | α-thujene |  | TR | 0.23 |
| 2 | 930 | 937 | | | α-pinene |  | 0.53 | 1.35 |
| 3 | 970 | 975 | | | sabinene |  | 0.43 | 1.11 |
| 4 | 974 | 975 | | | β-pinene | 5.5-9.5 | 3.58 | 9.41 |
| 5 | 988 | 989 | | | β-myrcene |  | 0.28 | 0.80 |
| 6 | 1022 | 1026 | | | paracymene |  | 0.50 | 1.23 |
| 7 | 1027 | 1029 | | | limonene | 30-45 | 16.96 | 43.72 |
| 8 | 1056 | 1062 | | | γ-terpinene | 6-10 | 2.90 | 7.39 |
| 9 | 1083 | 1088 | | | terpinolene |  | TR | 0.19 |
| 10 | 1103 | 1100 | | | linalool | 3-15 | 2.62 | 6.50 |
| 11 | 1125 | 1127 | | | methyl octanoate^i^ |  | 0.41 | 2.31 |
| 12 | 1192 | 1191 | | | α-terpineol* |  | TR | 0.12 |
| 13 | 1225 | 1253 | | | linalyl acetate | 22-36 | 10.03 | 26.35 |
| 14 | 1267 |  | | | geranial | 0.25-0.5 | TR | 0.15 |
| 15 | 1346 |  | | | hydroxy-acetophenone* |  | TR | 0.11 |
| 16 | 1358 | 1362 | | | neryl acetate |  | TR | 0.19 |
| 17 | 1377 | 1383 | | | geranyl acetate |  | TR | 0.16 |
| 18 | 1412 |  | | | β-caryophyllene |  | TR | 0.18 |
| 19 | 1428 |  | | | bergamotene* |  | TR | 0.15 |
| 20 | 1504 |  | | | β-bisabolene | 0.3-0.55 | 0.10 | 0.24 |
| 21 | 1625 |  | | | n-tetradecylamine* |  | 0.13 | -- |
| 22 | 1659 |  | | | triethyl citrate^ |  | 61.12 | -- |
|  | | |  | Notes: TR, Trace <%, --, not ID, *Tentative identification (similarity <85%), ^i^Internal standard, ^ Extraordinary effects. | | | | |
|  | | |  | All results the average of three sample injections, % area excludes area of Methyl Octanoate ISTD | | | | |

|  | |  | **Table 6:** Mandarin peak areas for samples with a-typical profiles | | | | | | | |
| --- | --- | --- | --- | --- | --- | --- | --- | --- | --- | --- |
| **#** | **RI** | **RI Lit** | **Compound** | **ISO** | **M1** | **M2** | **M9** | **M10** | **M11** |  |
|  |  |  |  | **range** | % | % | % | % | % |  |
| 1 | 923 | 923 | α-thujene |  | TR | -- | 0.69 | 0.12 | TR |  |
| 2 | 930 | 937 | α-pinene | 2-3 | 0.59 | 0.58 | 2.11 | 0.77 | 0.72 |  |
| 3 | 970 | 975 | sabinene |  | 0.36 | 1.07 | 0.27 | 0.13 | 0.28 |  |
| 4 | 974 | 975 | β-pinene | 1.2-2 | 0.12 | -- | 1.49 | 0.28 | 0.28 |  |
| 5 | 988 | 989 | β-myrcene | 1.5-2 | 1.76 | 1.41 | 1.69 | 1.66 | 1.42 |  |
| 6 | 1008 | 1018 | α-terpinene |  | -- | -- | -- | -- | 0.28 |  |
| 7 | 1022 | 1026 | paracymene |  | -- | -- | 0.26 | TR | TR |  |
| 8 | 1027 | 1029 | limonene | 65-75 | 96.22 | 95.90 | 76.44 | 93.73 | 74.05 |  |
| 9 | 1056 | 1062 | γ-terpinene | 16-22 | 0.44 | -- | 15.70 | 2.81 | 0.17 |  |
| 10 | 1083 | 1088 | terpinolene |  | TR | -- | 0.53 | 0.12 | TR |  |
| 11 | 1103 | 1100 | linalool^ |  | 0.18 | 0.28 | TR | 0.14 | 10.25 |  |
| 12 | 1125 | 1127 | methyl octanoate^i^ |  | 0.33 | 0.37 | 0.31 | 0.30 | 0.33 |  |
| 13 | 1132 |  | limonene 1,2-oxide |  | TR | 0.18 | TR | TR | 0.12 |  |
| 14 | 1148 |  | citronellal* |  | TR | 0.14 | TR | -- | 0.11 |  |
| 15 | 1194 | 1191 | α-terpineol |  | TR | TR | TR | TR | 0.24 |  |
| 16 | 1206 |  | decanal |  | TR | 0.16 | -- | -- | -- |  |
| 17 | 1225 | 1253 | linalyl acetate^ |  | -- | -- | -- | -- | 11.48 |  |
| 18 | 1245 |  | neral |  | -- | 0.17 | -- | -- | 0.12 |  |
| 19 | 1268 |  | methyl citronellate* |  | -- | -- | -- | TR | 0.15 |  |
| 20 | 1358 | 1362 | neryl acetate* |  | -- | -- | -- | TR | 0.11 |  |
| 21 | 1378 | 1383 | geranyl acetate* |  | -- | -- | -- | TR | 0.14 |  |
| 22 | 1402 | 1369 | methyl N-methyl anthranilate* | 0.3-0.6 | -- | -- | 0.46 | -- | -- |  |
| 23 | 1503 |  | α-farnesene* |  | TR | TR | TR | -- | -- |  |
| 24 | 1720 |  | α-sinensal* | 0.2-0.5 | TR | TR | TR | -- | -- |  |
|  |  |  |  |  |  |  |  |  |  |  |
|  | |  | Notes: TR, Trace <%, --, not ID, *Tentative identification (similarity <85%), ^i^Internal standard, ^ extraordinary effects | | | | | |  | |
|  | |  | All results the average of three sample injections, % area excludes area of Methyl Octanoate ISTD | | | | | |  | |

*Chromatogram*****

**Figure 1:** Select a-typical neroli chromatograms. Denoted by ^r^, the reference chromatogram represents a sample with expected complexity and response for comparison. Arrows refer to remarkable peaks: N1 is a peak of ~9% of the total area. N7 shows several broad peaks identified as DPG. N8 includes two arrows to mark the large peak at the start and the broad coelution caused by propylene glycol and glycerol respectively. N1 and N7 also have identified MS nerolin new (Figure 4).

**Figure 2:** A-typical bergamot chromatograms. Denoted by ^r^, the reference chromatogram represents a sample with expected complexity and response for comparison. Arrows refer to remarkable peaks: B1 triethyl citrate solvent peak comprising ~60% of the total peak area. B2 is heavily diluted in an “invisible” solvent, i.e. vegetal oil, and its chromatogram is only distinguishable from a typical sample by the low peak areas. The total peak area about 10 times lower than the other typical samples.

**Figure 3:** Select a-typical mandarin chromatograms. Denoted by ^r^, the reference chromatogram represents a sample with expected complexity and response for comparison. Arrows refer to remarkable peaks: M1 and M10 all have limonene representing >90% of the total peak area, uncharacteristic of mandarin. M10, purchased under a tangerine label conforms to a tangerine profile of >90% limonene and ~3% γ-terpinene (marked by arrow) as opposed to 16-22% γ-terpinene in mandarin. M11 contains linalool and linalyl acetate (marked by arrows).


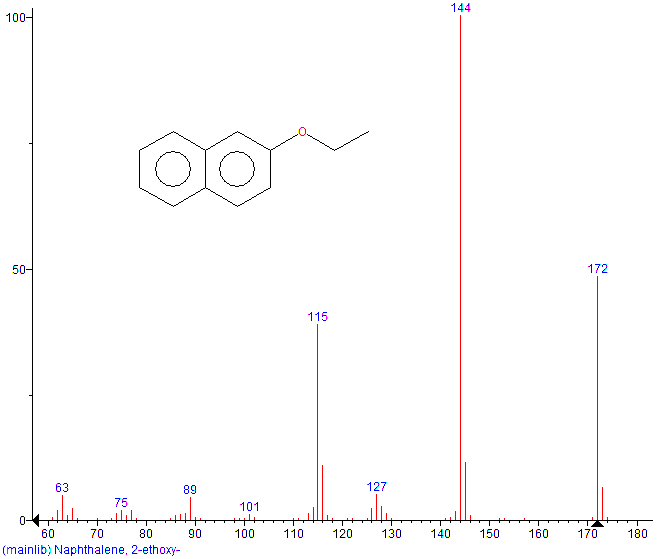


**Figure 4:** From NIST MS Search corresponding to the a-typical peak at RI 1519 in N1, N2 and N7 samples.

1. **NMR spectroscopy**

^1^H NMR spectra were recorded on BRUCKER AC spectrometer (400 MHz) in CDCl_3_ at 20 °C.

*Spectra*

B1

**Figure 5:** ^1^H NMR spectra of sample B1 with peaks corresponding to triethyl citrate circled.

B2

**Figure 6:** ^1^H NMR spectra of sample B2 with characteristic fatty oil peaks circled.

**Figure 7:** ^1^H NMR spectra of sample N1 with characteristic fatty oil peaks circled.

N1

1. **Graphical representation with heat Maps**

*Calculations*

To calculate the relative scores used to create the heat maps several steps are taken and described here. The terms in the equation for a statistical z score: $z=\frac{x-\mu}{\sigma}$ , were redefined as follows; µ (mean) becomes the midpoint of the AFNOR range for each regulated compound, σ (standard deviation) becomes the distance from the mid-point to the limit of the range. For example, the limonene specification in the mandarin AFNOR standard is 65-75% of the total area, making the µ=70 and the σ=5.

For each sample, the relative score of each compound in the AFNOR standard is calculated using the average of the triplicate results. The results are combined in a heatmap matrix. The color gradient of the matrix remains green from 0<z<1 then graduates from green to red from 1<z<1.8 with all amounts <1.8 set to the darkest red.

1. **HPLC analysis**

HPLC-PDA analysis was performed on reversed phase with a gradient from 98% Water to 98% Acetonitrile on a Teknokroma Sea18 column, 5mL/min and 310 nm detection. 5-methoxypsoralen standard was used as reference and injected at 34.4 ug/mL concentration.

Figure 8. Overlay of HPLC chromatograms of B1-B16 bergamot samples and 5-methoxypsoralen (zoom in the elution area of furocoumarins).

Figure 9. Overlay of HPLC chromatograms of M1, M2 and M10 mandarin samples, sweet orange essential oil and 5-methoxypsoralen (zoom in the elution area of furocoumarins).
